# Association Between Anti-Inflammatory Diet, Dietary Diversity, and Depressive Symptoms Among Chinese Pregnant Women

**DOI:** 10.3390/nu17172823

**Published:** 2025-08-29

**Authors:** Binyan Zhang, Ke Men, Chao Li, Kun Xu, Baibing Mi, Jiaxin Cai, Leilei Pei, Shunming Zhang, Yonghong Ma, Ying Li, Shaonong Dang, Hong Yan

**Affiliations:** 1 School of Public Health, Xi’an Medical University, Xi’an 710021, China; zhangbinyan@stu.xjtu.edu.cn (B.Z.); menke@xiyi.edu.cn (K.M.); mayhong292152@163.com (Y.M.); liying@xiyi.edu.cn (Y.L.); 2 Department of Epidemiology and Biostatistics, School of Public Health, Xi’an Jiaotong University Health Science Center, Xi’an 710061, China; lcxjtu@xjtu.edu.cn (C.L.); xjtu.mi@xjtu.edu.cn (B.M.); peileilei424@163.com (L.P.); 3 Department of Nutrition and Food Hygiene, School of Public Health, Tongji Medical College, Huazhong University of Science and Technology, Wuhan 430030, China; xukun_728@hust.edu.cn; 4 Complex Systems Research Center, Shanxi University, Taiyuan 030006, China; mathcjx@sxu.edu.cn; 5 School of Public Health, Xi’an Jiaotong University Health Science Center, Xi’an 710061, China; zhangshunming@xjtu.edu.cn; 6 Key Laboratory of Environment and Gene-Related Diseases, Xi’an Jiaotong University, Ministry of Education, Xi’an 710061, China

**Keywords:** anti-inflammatory diet, dietary diversity, depressive symptoms, pregnancy

## Abstract

**Background**: Dietary inflammatory potential and dietary diversity during pregnancy may program depression, yet these associations remain poorly characterized. This study aimed to assess an anti-inflammatory diet and dietary diversity in relation to depressive symptoms. **Methods**: We analyzed data from 2244 pregnant women in the prospective longitudinal study. Depressive symptoms were defined as an Edinburgh Postnatal Depression Scale (EPDS) score ≥ 9.5. An anti-inflammatory diet was assessed using the reverse energy-adjusted Dietary Inflammatory Index (rEDII), derived from food frequency questionnaires. Dietary diversity was classified as either adequate or inadequate. Generalized estimating equations were performed. **Results**: Our findings demonstrated that a higher rEDII was associated with lower EPDS scores (β: −0.25; 95% CI: −0.37, −0.12) and a 13% reduction in the risk of depressive symptoms (RR: 0.87; 95% CI: 0.81, 0.93). Adequate dietary diversity was linked to a 22% lower risk of depressive symptoms (RR: 0.78; 95% CI: 0.64, 0.95). A significant interaction was observed between rEDII and dietary diversity in relation to depressive symptoms. Among women with inadequate dietary diversity, a higher rEDII was associated with a 15% reduction in depressive symptom risk (RR: 0.85; 95% CI: 0.80, 0.90). **Conclusions**: An anti-inflammatory diet was associated with a lower risk of depressive symptoms; this association was greater among women with inadequate dietary diversity.

## 1. Introduction

Mental health disorders have become a significant global health crisis [1], impacting approximately 30% of individuals during their lifetime and ranking as the seventh leading cause of disability-adjusted life-years (DALYs) [2]. Among these disorders, depression is of particular concern as it is the top contributor to DALYs [3]. Notably, women, especially pregnant women, are more prone to depressive symptoms than men [4,5]. Perinatal depression, defined symptomatically as exceeding a threshold on a screening measure, such as the Edinburgh Postnatal Depression Scale [6], is associated with a range of adverse maternal health [7,8] and pregnancy outcomes [9,10], intergenerational negative impacts such as psychotic experiences in adults [11], behavioral disturbances later in life [12], and subsequent offspring depression [13,14]. Globally, the prevalence of perinatal depression is estimated at 20.7%, with significant disparities across economic regions. This prevalence is substantially higher in low- and lower-middle-income countries (24.2–30.3%) compared to high-income countries (18.1%) [15]. China mirrors this global pattern, demonstrating an urban-rural gradient in prevalence rates. While 18.4% of pregnant women in Guangzhou (an urban center) experience depression during early pregnancy [16], rural populations bear an even greater burden, with Shaanxi Province reporting a 19.2% prevalence [17]. These persistent disparities, particularly in resource-limited settings, necessitate targeted public health interventions to mitigate the impact of perinatal depression.

Current evidence suggests that inflammation may contribute to the pathophysiology of depression [18]. Randomized controlled trials and meta-analyses demonstrate that anti-inflammatory agents are effective in treating depression, supporting a potential causal relationship [19,20]. However, the use of these agents may be associated with adverse outcomes, limiting their widespread application [21]. Dietary interventions offer a promising non-pharmacological alternative, as diets are rich in bioactive compounds with pro- or anti-inflammatory properties [22]. Supplementation with minerals such as selenium, zinc, magnesium, or lithium is often employed as an adjunctive treatment for depression. Conversely, excessive fat intake may increase the risk of depression, potentially through inflammatory mechanisms [23]. In addition, a higher ratio of non-high-density lipoprotein cholesterol (non-HDL-C) to high-density lipoprotein cholesterol (HDL-C) has been significantly associated with a greater risk of depression [24]. Evidence suggests that reduced HDL-C levels can activate inflammatory pathways and thereby facilitate disease progression [25]. The “Food is Medicine” concept has gained recognition as a potential depression prevention strategy, given the growing evidence linking dietary inflammatory potential to depression risk [26,27,28,29]. Importantly, beyond inflammatory potential, adequate dietary diversity is protective against depression in adults [30], likely due to their role in promoting micronutrient adequacy and balance [31,32]. Nevertheless, it is important to note that nearly all available evidence regarding the associations of dietary inflammatory potential and dietary diversity with depression is derived from cross-sectional studies of general populations in developed nations [26,27,28,29,30]. Women of reproductive age are susceptible to the adverse effects of poor nutrition on mood [33]. Notably, *n*-3 polyunsaturated fatty acid intake has been implicated in brain function [34], and the neurobiological mechanisms of depression may be nutritionally modulated [18,35]. An emerging question is whether these associations generalize to pregnant women in rural China, a population characterized by a high depression burden (19.2%), pro-inflammatory dietary patterns, and inadequate dietary diversity [17,36,37].

This study aimed to investigate the associations of the anti-inflammatory diet and dietary diversity during pregnancy with depressive symptoms. We hypothesized that inadequate dietary diversity would amplify the pro-inflammatory effects of diet, thereby increasing depression risk. Accordingly, we examined how such associations between an anti-inflammatory diet and depressive symptoms might be modified by dietary diversity to inform targeted nutritional intervention strategies for Chinese pregnant women.

## 2. Materials and Methods

### 2.1. Study Design and Participants

The original design of this study was a community-based randomized controlled trial (registered at ClinicalTrials.gov: NCT02537392, https://clinicaltrials.gov/study/NCT02537392 (accessed on 4 August 2025)) conducted to evaluate the effect of multivitamin supplementation during pregnancy on birth outcomes. The details of the trial and primary findings have been published previously [38]. Briefly, the trial was implemented across three rural counties in Shaanxi Province, China, between July 2015 and December 2019. We enrolled pregnant women aged 15–47 years with a gestational age < 20 weeks at recruitment who planned to maintain residence in the study area for one year postpartum. Using township-level randomization to ensure geographical balance, participants were allocated in a 1:1:1 ratio to three intervention arms: 400 μg folic acid supplement alone, 400 μg folic acid plus 60 mg iron, or 400 μg folic acid with B-complex vitamins. The B-complex vitamins consisted of 2 mg vitamin B_1_, 2 mg vitamin B_2_, 2 mg vitamin B_6_, 2 μg vitamin B_12_, 5 mg calcium pantothenate, and 15 mg nicotinamide. For the current analysis, we included participants with complete prenatal dietary assessments. Those who were lost to follow-up, withdrew from the study, experienced pregnancy termination (abortion or stillbirth), had hypertension or diabetes, or reported extreme energy intake (<500 or >5000 kcal/day) were excluded [39,40].

The study protocol was conducted in accordance with the principles of the Declaration of Helsinki and received ethical approval from the Ethics Review Committee of Xi’an Jiaotong University Health Science Center (No. 20120008). Written informed consent was obtained from all participants after they received a comprehensive explanation of the study objectives and procedures.

### 2.2. Maternal Dietary Assessment

Dietary intake was assessed using a validated food frequency questionnaire (FFQ) administered biweekly during pregnancy. This FFQ demonstrated good reproducibility and validity for evaluating nutrient intake among rural Chinese pregnant women, showing mean Pearson correlation coefficients of 0.62 (range: 0.53–0.70) when compared with 24 h recall data for all nutrients [41]. The FFQ consisted of 102 specified food items as well as additional questions regarding vegetable oil, animal oil, salt, sauce, and sugar. A full list of food items is provided in Table S1. For each food item, participants were asked to report the frequency and portion size of consumption during pregnancy. Frequency was assessed using an eight-category scale, ranging from “almost never” to “2 or more times” per day. Portion sizes were quantified based on the small, medium, and large portion sizes depicted in a food atlas, with corresponding gram weights assigned to each portion size.

### 2.3. Assessment of Maternal Dietary Inflammatory Potential

The Dietary Inflammatory Index (DII) was a well-validated literature-derived score, which was applicable across diverse populations [42]. The DII was originally developed based on 45 dietary components. In this study, the DII was assessed using 33 food parameters from dietary surveys conducted in rural areas. The calculation process involved four key steps. First, energy and nutrient intakes were quantified according to the Chinese Food Composition Table [43,44]. Second, standardized z-scores were computed for each dietary component by first deducting the global average intake from individual reported intakes and then dividing the result by the global standard deviation. Third, these z-scores were converted to percentile values, which were then centered by doubling and subtracting one, followed by multiplication with the corresponding component-specific inflammatory weight. The final DII score represented the summation of all component-specific values obtained through this transformation process. The reverse energy-adjusted DII (rEDII) was calculated to quantify anti-inflammatory dietary potential [45].

### 2.4. Derivation of Dietary Diversity Score

Dietary diversity score (DDS) was derived from the Minimum Dietary Diversity for Women (MDD-W) guidelines [46], a validated measure for low- and middle-income populations [47]. Following the FAO framework, we evaluated consumption across ten defined food groups: starchy staples, beans and peas, nuts and seeds, dairy, flesh foods, eggs, vitamin A-rich dark green vegetables, other vitamin A-rich fruits and vegetables, other vegetables, and other fruits. Food group intake was assessed relative to the recommended thresholds specified in the Chinese Dietary Guidelines (2022). For each food group, participants received a score of 1 if their consumption met the guideline recommendations and 0 if it did not. These scores were summed to produce a total DDS ranging from 0 to 9, which was subsequently dichotomized to classify participants into either the adequate dietary diversity group (DDS ≥ 5) or the inadequate dietary diversity group (DDS < 5).

### 2.5. Measurement of Depressive Symptoms

Depressive symptoms were assessed during the third trimester of pregnancy (mean gestational age, 38.7 ± 1.2 weeks) using the Edinburgh Postnatal Depression Scale (EPDS). Although originally developed for postpartum depression screening, the EPDS has been validated for and is widely employed in the assessment of prenatal depressive symptoms [6,48]. This 10-item scale scores each item from 0 to 3, resulting in a total score range of 0–30. Higher total scores reflected greater depressive symptom severity. A cut-off score of ≥9.5 was used to indicate a positive screen, with a sensitivity of 82% and a specificity of 86% for diagnosing depression [49].

### 2.6. Measurement of Covariates

Covariates were selected based on recent evidence regarding potential determinants of depression and their dietary associations [50,51,52]. Of these, multivitamin supplements were categorized into folic acid alone, folic acid plus iron, and folic acid plus B-complex vitamins. Maternal age was analyzed as a continuous variable. Parity was dichotomized into nulliparous and multiparous categories. Socioeconomic status was constructed through principal component analysis of five indicators, including household wealth index, parental education, and parental occupation. Participants were then categorized into lower, middle, and upper tertiles. Body mass index (BMI, kg/m^2^) at enrollment was calculated as weight in kilograms divided by height squared in meters. Pregnancy complications were defined as clinically diagnosed pregnancy-induced hypertension or gestational diabetes mellitus.

### 2.7. Statistical Analysis

Analyses were conducted using SAS version 9.4 (SAS Institute, Cary, NC, USA), with a significance level set at α = 0.05. Baseline characteristics of the study population, stratified by rEDII quartiles and DDS classification, were summarized as frequencies and percentages for categorical variables and means ± standard deviations (SDs) for continuous variables. To account for potential clustering effects by county, significance tests were performed using generalized estimating equations (GEE). Similarly, mean differences in food group and food parameter intakes across rEDII quartiles and DDS classifications were assessed using GEE, with adjustments made for county-level clustering.

GEE was also built to estimate the β coefficients or risk ratios (RRs) and the 95% confidence intervals (95% CIs) for the associations of anti-inflammatory diet and adequacy of DDS during pregnancy with EPDS scores and the incidence of depressive symptoms. Three GEE models were fitted with incremental adjustment for potential confounders. Model 1 was a crude model. Model 2 added multivitamin supplements (folic acid alone, folic acid plus iron, and folic acid plus B-complex vitamins) on the basis of Model 1. Model 3 adjusted for maternal age (continuous), nulliparity status (nulliparous or multiparous), socioeconomic situation (lower, medium, and upper), passive smoking (yes or no), and BMI at enrollment (continuous) on the basis of Model 2. Clustering by county was accounted for in all analyses using GEEs to account for non-independent data within each county.

To evaluate whether DDS modified the association of interest, we examined potential effect modification by adequate or inadequate DDS on the associations of rEDII with changes in EPDS scores or the risk of incident depressive symptoms. Specifically, we introduced multiplicative interaction terms (rEDII × DDS group) into fully adjusted models to examine the extent of DDS-mediated modification. Furthermore, stratified analysis of the association of dietary inflammatory potential with EPDS scores or depressive symptoms by DDS group was summarized with β coefficients or RRs and the 95% CIs. These estimates were derived from GEEs with an identity or log-binomial link function.

## 3. Results

This prospective longitudinal study recruited 2438 pregnant women with dietary assessments from rural China between July 2015 and December 2019. After excluding those who were lost to follow-up (*n* = 70, 2.9%), withdrew (*n* = 16, 0.7%), had fetal loss (*n* = 20, 0.8%) or stillbirth (*n* = 4, 0.2%), had hypertension or diabetes (*n* = 24, 1.0%), missing depression data (*n* = 32, 1.3%), and had extreme dietary intake (*n* = 28, 1.1%), there were 2244 women included in the analysis (Figure S1). The mean ± SD gestational age at recruitment into the study was 13.6 ± 4.6 weeks. Dietary assessment was conducted at a mean gestational age of 26.2 ± 12.4 weeks. Although on average the dietary assessment was conducted at 26.2 weeks, the diet was always associated temporally prior to the occurrence of depressive symptoms assessed at 38.7 weeks. Mean ± SD gestational age at birth in the study was 39.7 ±1.2 weeks. EDII was −0.00 ± 1.81 units with a range of −5.18 to 7.39. DDS scores for women ranged from 0 to 9, with a median of 3.0 (IQR: 2.0–4.0) during pregnancy. Only 443 women (19.7%) met the adequate dietary diversity (DDS ≥ 5). A Spearman correlation analysis revealed an inverse association (*r* = −0.36, *p* < 0.001) between EDII and adequate DDS.

Table 1 presents the baseline characteristics of the study population. There were no significant differences in assignment to folic acid plus multivitamins or folic acid alone in either rEDII or DDS groups. The distribution of parity and pregnancy complications was similar across quartiles of the rEDII as well as inadequate and adequate DDS. When comparing women in the lowest quartile (Q1) with those in the highest quartile (Q4) of rEDII, those in Q4 were older, more likely to belong to an upper socioeconomic status, had lower exposure to passive smoking, and showed a lower prevalence of depressive symptoms (*p* < 0.05). Regarding dietary diversity, women with inadequate DDS had higher EDII scores, were more likely to be of medium socioeconomic status, had higher BMI, and exhibited more depressive symptoms compared to those with adequate DDS (*p* < 0.05). However, no significant differences were found in maternal age or passive smoking exposure between the inadequate and adequate DDS.

Table 2 shows the distribution of food groups across quartiles of rEDII and DDS groups. Participants in Q4 of rEDII had higher consumption of miscellaneous beans, tuber crops, vegetables/fruit, red meat, white meat, organ meat, fish/seafood, whole eggs, milk/legumes and nuts, and snacks. In contrast, the consumption of grains, vegetable oils, animal oils, soy sauce, and white/brown sugar was found to be lower in Q4 of rEDII. Participants in the adequate DDS group consumed greater amounts of grains and tuber crops, vegetables/fruit, animal-based food, milk/legumes and nuts, vegetable oils, soy sauce, white/brown sugar, snacks, and soft drinks. Among all pregnant women, starchy staples were nearly universally consumed (98.2%), followed by eggs (60.9%) and other vitamin A-rich fruits and vegetables (56.9%). In contrast, the consumption of flesh foods (1.5%), nuts and seeds (1.7%), other vegetables (5.0%), as well as beans and peas (11.3%) was lower. Notably, women with inadequate dietary diversity (DDS < 5) consumed greater quantities of starchy staples, dairy, eggs, other vitamin A-rich fruits and vegetables, and other fruits. Conversely, beans and peas, nuts and seeds, flesh foods, vitamin A-rich dark green vegetables, and other vegetables showed higher intake in the adequate DDS group (Table S2).

As presented in Table 3, participants with both the highest rEDII quartile and adequate DDS demonstrated significantly higher consumption of energy, protein, fat, cholesterol, fiber, vitamins (except for Vitamin B_12_), minerals, isoflavones, anthocyanidins, onions, and garlic. Notably, carbohydrate intake remained higher in the adequate DDS group compared to the inadequate DDS group.

The overall incidence of depressive symptoms was 10.6% (*n* = 237), with 11.1% (*n* = 199) in the inadequate DDS group and 8.6% (*n* = 38) in the adequate group. In multivariate analysis, we found an inverse association between rEDII and both EPDS scores (β: −0.25; 95% CI: −0.37, −0.12) and the risk of depressive symptoms (RR: 0.87; 95% CI: 0.81, 0.93). Women in the lowest quartile of rEDII during pregnancy demonstrated a 0.50 reduction in EPDS scores (β: −0.50; 95% CI: −0.88, −0.13) and a 25% lower risk of depressive symptoms (RR: 0.75; 95% CI: 0.58, 0.97) in fully adjusted models. While no significant association emerged between DDS groups and EPDS scores (β: −0.39; 95% CI: −0.81, 0.03), adequate DDS was associated with a 22% reduction in depressive symptoms’ risk (RR: 0.78; 95% CI: 0.64, 0.95) (Table 4).

Table 5 shows that there were significant interactions between rEDII and DDS groups in relation to EPDS scores (*p* for interaction < 0.001) and depressive symptoms (*p* for interaction = 0.004). Associations of rEDII with EPDS scores and depressive symptoms stratified by DDS groups are detailed in Table 6. After adjusting for potential covariates, the inverse association between rEDII and EPDS scores was more pronounced in the inadequate DDS group (β: −0.24; 95% CI: −0.32, −0.16) than in the adequate DDS group (β: −0.10; 95% CI: −0.14, −0.05). Among women with inadequate DDS, higher rEDII was associated with a 15% reduction in the risk of depressive symptoms (RR: 0.85; 95% CI: 0.80, 0.90). In contrast, pregnant women with adequate DDS showed no significant associations between rEDII (RR: 1.20; 95% CI: 0.97, 1.50) and depressive symptoms.

## 4. Discussion

To our knowledge, this is the first prospective study to concurrently evaluate the role of an anti-inflammatory diet and dietary diversity against perinatal depressive symptoms among pregnant women in rural China. Our analyses demonstrate that higher adherence to an anti-inflammatory diet and adequate dietary diversity during pregnancy were associated with a reduced risk of depressive symptoms. We also observe a significant interaction between the anti-inflammatory diet and dietary diversity on depressive symptoms, suggesting that inadequate dietary diversity may benefit more from anti-inflammatory diets.

Our findings are supported by longitudinal studies from high-income countries, including Australia [29], the United States [53], and the United Kingdom [54], where pro-inflammatory diets have been consistently associated with an increased risk of depressive symptoms in general adult populations. However, the observed sex-specific variations in dietary inflammatory potential warrant particular attention. Notably, a French cohort study with a mean baseline age of 52.1 years found no overall association between pro-inflammatory diets and incident depressive symptoms in the general population, though a significant association emerged in males [55]. Findings from an Australian longitudinal study with a mean baseline age of 52.0 years showed that an anti-inflammatory diet was associated with a reduced depression risk in middle-aged women [56]. Pregnancy is a unique biological state characterized by immunological adaptations and hormonal fluctuations that may amplify vulnerability to dietary inflammatory effects [57,58]. Nonetheless, prospective epidemiological evidence investigating this association during pregnancy remains limited. To date, only the Guangzhou Cohort Study has reported an inverse association between high consumption of fruits and vegetables throughout pregnancy and the incidence of depressive symptoms in pregnant women [59]. When integrated with randomized controlled trial evidence that increased fruit and vegetable intake reduced plasma *C*-reactive protein concentrations, these findings collectively suggested that an anti-inflammatory diet might alleviate maternal depressive symptoms through modulation of inflammatory pathways [60].

Furthermore, we demonstrated that adequate dietary diversity was significantly associated with a reduced risk of depressive symptoms, aligning with existing evidence [30,61,62]. Meanwhile, evidence from both cross-sectional and longitudinal research suggested that greater DDS might play a key role in maintaining a low inflammatory status [63]. This observation could partially explain the relationship between dietary diversity and depressive symptoms, as adequate dietary diversity may mitigate depressive risk by reducing chronic maternal inflammation. In addition, we found that adequate DDS was characterized by greater consumption of beans and peas, nuts and seeds, meats, and vegetables, which was inversely associated with the EDII. This dietary pattern closely aligns with the anti-inflammatory properties of the Mediterranean diet, which had a protective influence against depressive symptoms [64,65]. The result that women with inadequate DDS benefited more from anti-inflammatory diets further supports the finding.

There are several mechanisms underlying the potential protective role of an anti-inflammatory diet against the risk of depressive symptoms. One is the brain-gut-microbiota axis [66], a bidirectional communication system that integrates gastrointestinal and central nervous system function, playing a critical role in neuropsychiatric disorders, including depression [67,68]. Diets serve as a primary modifiable regulator of this axis, with pro-inflammatory diets altering gut microbial composition, diversity, and metabolic activity [69]. Such changes may affect neuronal function and synaptic plasticity, thereby increasing susceptibility to depression. Moreover, pro-inflammatory diets can promote chronic immune system activation, establishing a state of low-grade systemic inflammation. This process involves the activation of immune cells, particularly polymorphonuclear leukocytes, which generate excessive reactive oxygen species (ROS). The resulting oxidative stress can damage brain neurons and contribute to the development of depression [50]. Furthermore, ROS can also modulate inflammatory responses by activating key transcription factors such as nuclear factor-kappa B (NF-κB) and activator protein-1 (AP-1), thereby upregulating pro-inflammatory cytokine expression [70,71]. These cytokines, in turn, can dysregulate the hypothalamic-pituitary-adrenal (HPA) axis, leading to hippocampal volume reduction, impaired neuronal plasticity, and decreased neurochemical functioning, resulting in depressive symptoms [72].

Our findings may have important public health implications. The results highlight the importance of a maternal anti-inflammatory diet and dietary diversity during pregnancy in relation to maternal mental health. However, it should not be assumed that dietary intake during pregnancy drives the observed association. Given that diet often requires a sustained period to exert effects on maternal mental health, the association is likely attributable to the diet prior to pregnancy, which has been proven to be strongly associated with the diet during pregnancy [73,74]. In addition, prepregnancy and pregnancy diets are related to maternal mental health and long-term offspring health. Therefore, enhancing awareness of maternal nutrition, ensuring access to affordable, diverse, and anti-inflammatory foods, and providing early professional guidance would help women achieve a healthier diet and promote maternal and child health.

This study has several strengths. This is the first prospective longitudinal study to simultaneously examine the associations of a maternal anti-inflammatory diet and dietary diversity with depressive symptoms among pregnant women in rural China. Since dietary assessment occurred prior to depressive symptoms’ identification, the temporal relationship partially supports a causal interpretation. There are several limitations of the study that must be considered. First, although we adjusted for nutrient interventions in the randomized controlled trial, the generalizability of the findings is still limited. Second, both the dietary information and depressive symptoms status were self-reported by the participants, which might increase nondifferential measurement errors that might bias results toward the null. Nonetheless, the FFQ and EPDS have been proven to be valid and reliable in epidemiologic studies [10,36]. Third, although our analysis employed 33 of the 45 originally proposed food parameters to calculate the DII score, the absence of data for the remaining 12 food parameters might be a potential limitation. However, previous studies have demonstrated that the predictive power of DII was not reduced when using fewer than 30 dietary parameters [75]. Fourth, potential residual confounding could not be ruled out due to unmeasured factors associated with both diet and depressive symptoms, such as a history of depression or lack of social support [15,16].

## 5. Conclusions

Our findings demonstrate that an anti-inflammatory diet was associated with a lower risk of depressive symptoms; this association was greater among pregnant women with inadequate dietary diversity. These findings highlight that adherence to an anti-inflammatory diet and adequate dietary diversity can be an effective non-pharmacological preventive strategy for depressive symptoms.

## Figures and Tables

**Table 1:** Baseline characteristics of study population by quartiles of reverse energy-adjusted dietary inflammatory index and dietary diversity during pregnancy in rural China, 2015–2019.

Baseline Characteristics	*n* = 2244	Quartiles of rEDII				*p*	DDS Groups		*p*
		Q1 (*n* = 561)	Q2 (*n* = 561)	Q3 (*n* = 561)	Q4 (*n* = 561)		Adequate (*n* = 443)	Inadequate (*n* = 1801)	
Multivitamin supplement						0.113			0.504
FA	814(36.3)	201(35.8)	210(37.4)	190(33.9)	213(38.0)		158(35.7)	656(36.4)	
FA + iron	704(31.4)	162(28.9)	178(31.7)	190(33.9)	174(31.0)		135(30.5)	569(31.6)	
FA + B-complex vitamins	726(32.4)	198(35.3)	173(30.8)	181(32.3)	174(31.0)		150(33.9)	576(32.0)	
EDII	−0.00 ± 1.81	2.32 ± 0.88	−0.63 ± 0.37	0.62 ± 0.40	−2.30 ± 0.71		−1.30 ± 1.55	0.32 ± 1.72	<0.001
EDII range	−5.18, 7.39	1.29, 7.39	−1.30, −0.02	−0.02, 1.29	−5.18, −1.30		−5.18, 4.69	−4.27, 7.39	
Age (years)	25.8 ± 4.1	25.4 ± 4.2	25.9 ± 4.2	25.7 ± 4.0	26.3 ± 3.9	<0.001	26.0 ± 4.1	25.8 ± 4.1	0.505
Parity						0.174			0.720
Nulliparous	1095(48.8)	271(48.3)	251(44.7)	284(50.6)	289(51.5)		220(49.7)	875(48.6)	
Multiparous	1149(51.2)	290(51.7)	310(55.3)	277(49.4)	272(48.5)		223(50.3)	926(51.4)	
Socioeconomic status ^1^						<0.001			0.027
Lower	747(33.3)	208(37.1)	180(32.1)	193(34.4)	166(29.6)		150(33.9)	597(33.2)	
Medium	747(33.3)	196(34.9)	189(33.7)	183(32.6)	179(31.9)		128(28.9)	619(34.4)	
Upper	750(33.4)	157(28.0)	192(34.2)	185(33.0)	216(38.5)		165(37.3)	585(32.5)	
Body mass index (kg/m^2^)	21.4 ± 2.7	21.5 ± 3.0	21.5 ± 2.5	21.5 ± 2.7	21.3 ± 2.5	0.061	21.3 ± 2.5	21.5 ± 2.7	0.033
Passive smoking ^2^	248(11.1)	64(11.4)	71(12.7)	62(11.1)	51(9.1)	<0.001	50(11.3)	198(11.0)	0.681
Pregnancy complication ^3^	70(3.1)	22(3.9)	22(3.9)	12(2.1)	14(2.5)	0.065	14(3.2)	56(3.1)	0.953
Depressive symptoms						0.012			0.007
No (EPDS < 9.5)	2007(89.4)	490(87.3)	502(89.5)	505(90.0)	510(90.9)		405(91.4)	1602(89.0)	
Yes (EPDS ≥ 9.5)	237(10.6)	71(12.7)	59(10.5)	56(10.0)	51(9.1)		38(8.6)	199(11.1)	

rEDII, reverse energy-adjusted dietary inflammatory index; DDS, dietary diversity score; FA, folic acid; EPDS, Edinburgh Postnatal Depression Scale. Values are mean ± standard deviations or n (%). Tests of significance are adjusted for clustering by county using a generalized estimating equation. ^1^ Socioeconomic status was assessed using principal component analysis to derive a composite score from five indicators: family wealth index, maternal and paternal education levels, and maternal and paternal occupations. Socioeconomic status was then categorized into lower, medium, and higher groups based on tertiles of the composite score. ^2^ Passive smoking was defined as exposure to second-hand smoke for >15 min per day during pregnancy. ^3^ Pregnancy complications included pregnancy-induced hypertension syndrome and gestational diabetes mellitus.

**Table 2:** Food groups consumed by quartiles of reverse energy-adjusted dietary inflammatory index and dietary diversity score among pregnant women in rural China, 2015–2019.

Food Groups	Quartiles of rEDII		DDS Groups	
	Q1	Q4	Adequate	Inadequate
Grains and tuber crops				
Grains (g/d)	876.9 ± 340.4	772.2 ± 197.3 ***	918.1 ± 278.3	802.8 ± 271.6 ***
Miscellaneous beans (g/d)	3.0 ± 5.8	6.3 ± 8.0 ***	6.7 ± 7.9	3.9 ± 6.3 ***
Tuber crops (g/d)	40.3 ± 38.5	78.9 ± 53.6 ***	86.2 ± 55.1	54.0 ± 43.6 ***
Vegetables/fruit (g/d)				
Leafy green vegetables (g/d)	100.6 ± 73.6	263.2 ± 135.0 ***	295.6 ± 140.3	145.6 ± 92.1 ***
Dark yellow vegetables (g/d)	45.6 ± 43.3	97.2 ± 51.4 ***	107.0 ± 50.2	64.4 ± 48.7 ***
Dark purple vegetables (g/d)	14.1 ± 18.3	38.4 ± 32.8 ***	43.7 ± 35.5	20.6 ± 23.1 ***
Light white vegetables (g/d)	27.9 ± 27.9	81.9 ± 60.1 ***	97.4 ± 69.9	41.5 ± 33.5 ***
Fruit (g/d)	345.7 ± 397.7	686.8 ± 423.1 ***	741.6 ± 493.2	456 ± 354.8 ***
Animal-based food (g/d)				
Red meat (g/d)	11.2 ± 17.7	16.7 ± 21.2 ***	24.1 ± 28.3	11.8 ± 15.5 ***
White meat (g/d)	3.6 ± 6.6	5.6 ± 10.0 ***	7.8 ± 13.0	3.6 ± 5.6 ***
Processed meat (g/d)	7.4 ± 16.0	7.1 ± 10.8 ***	11.3 ± 19.5	6.8 ± 13.0 ***
Organ meat (g/d)	0.4 ± 2.4	1.6 ± 6.2 ***	2.0 ± 7.4	0.7 ± 2.7 ***
Fish/seafood (g/d)	4.5 ± 8.0	9.0 ± 11.9 ***	11.2 ± 14.6	5.3 ± 7.2 ***
Whole eggs (g/d)	22.6 ± 22.2	31.9 ± 24.0 ***	39.0 ± 22.7	25.0 ± 22.6 ***
Milk/legumes and nuts (g/d)				
Milk and dairy products (g/d)	86.8 ± 102.3	130.6 ± 112.3 ***	170.9 ± 135.1	96.7 ± 103.0 ***
Legumes and nuts (g/d)	85.3 ± 156	241.3 ± 128.7 ***	253.3 ± 159.2	139.5 ± 141.9 ***
Condiments (g/d)				
Vegetable oils (g/d)	31.5 ± 6.3	30.0 ± 5.9 *	31.4 ± 5.6	30.7 ± 6.1 ***
Animal oils (g/d)	4.0 ± 6.5	1.6 ± 4.5 ***	2.9 ± 5.8	2.8 ± 5.7
Salt (g/d)	7.4 ± 1.6	7.4 ± 1.7	7.4 ± 1.6	7.4 ± 1.6
Soy sauce (g/d)	5.1 ± 3.9	4.5 ± 3.4 ***	5.1 ± 3.6	4.7 ± 3.7 *
White/brown sugar (g/d)	3.9 ± 3.2	3.1 ± 3.0 **	3.7 ± 3.1	3.5 ± 3.1 ***
Snacks/drinks (g/d)				
Snacks (g/d)	7.8 ± 11.8	9.3 ± 10.9 **	12.5 ± 15	8.5 ± 12.0 ***
Soft drinks (g/d)	7.7 ± 46.0	9.3 ± 44.0	8.1 ± 31.8	6.9 ± 34.7 ***
Alcohol drinks (g/d)	0.6 ± 11.0	0.1 ± 1.0	0.1 ± 1.0	0.3 ± 6.2

rEDII, reverse energy-adjusted dietary inflammatory index; DDS, dietary diversity score; Q, quartile. Values are means ± standard deviations. *p* values are from generalized estimating equations adjusting for clustering by county. *** *p* < 0.001, ** *p* < 0.01, * *p* < 0.05.

**Table 3:** Food parameters consumed by quartiles of reverse energy-adjusted dietary inflammatory index and dietary diversity score during pregnancy in rural China, 2015–2019.

Food Parameters	Quartiles of rEDII		DDS Groups	
	Q1	Q4	Adequate	Inadequate
Energy (kcal/d)	2244.0 ± 878.0	2340.2 ± 461.6 **	2832.1 ± 691.8	2163.1 ± 651.8 ***
Carbohydrate (g/d)	374.8 ± 167.2	358.9 ± 82.4	448.2 ± 127.6	346.9 ± 123.9 ***
Protein (g/d)	59.7 ± 26.4	71.7 ± 17.6 ***	86.9 ± 23.2	60.4 ± 20.2 ***
Total fat (g/d)	63.4 ± 27.0	76.6 ± 18.4 ***	86.6 ± 24.1	66.3 ± 21.9 ***
Saturated fat (g/d)	14.2 ± 7.7	16.2 ± 5.7 ***	21.0 ± 7.8	14.1 ± 6.1 ***
MUFA (g/d)	28.1 ± 8.2	30.7 ± 6.4 ***	35.0 ± 8.4	28.2 ± 6.8 ***
PUFA (g/d)	15.1 ± 13.6	24.6 ± 8.8 ***	24.5 ± 10.3	18.5 ± 11.3 ***
*n*-3 fatty acids (g/d)	4.2 ± 5.4	7.6 ± 3.5 ***	6.9 ± 3.9	5.5 ± 4.5 ***
*n*-6 fatty acids (g/d)	15.0 ± 13.6	24.9 ± 8.8 ***	24.8 ± 10.4	18.5 ± 11.4 ***
Cholesterol (mg/d)	178 ± 141.2	243.8 ± 146.7 ***	311.1 ± 146.6	190.6 ± 136.1 ***
Fiber (g/d)	7.1 ± 5.2	14.7 ± 4.6 ***	16.3 ± 5.8	9.5 ± 4.8 ***
Vitamins				
Vitamin A (μg/d, RAE)	235.2 ± 212.6	503.3 ± 372.6 ***	584.3 ± 391	300.4 ± 205.1 ***
β-Carotene (μg/d)	1151.7 ± 872.3	3306.4 ± 1432.0 ***	3555.9 ± 1588.6	1805.6 ± 1125.5 ***
Thiamin (mg/d)	0.8 ± 0.4	1.0 ± 0.2 ***	1.2 ± 0.3	0.8 ± 0.3 ***
Riboflavin (mg/d)	0.9 ± 0.7	1.2 ± 0.4 ***	1.5 ± 0.7	0.9 ± 0.5 ***
Niacin (mg/d)	11.4 ± 4.9	15.3 ± 4.2 ***	18.2 ± 5.3	12.2 ± 4.0 ***
Vitamin B_6_ (mg/d)	1.0 ± 0.6	1.7 ± 0.5 ***	1.9 ± 0.6	1.2 ± 0.5 ***
Vitamin B_12_ (μg/d)	4.9 ± 5.2	4.1 ± 4.6 ***	6.4 ± 5.3	4.0 ± 4.1 ***
Folic acid (μg/d)	184.3 ± 111.4	399.8 ± 120.8	446.9 ± 141.5	248.8 ± 111.8 ***
Vitamin C (mg/d)	68.0 ± 44.0	164.8 ± 57.0 ***	179.3 ± 67.9	98.6 ± 50.6 ***
Vitamin D (μg/d)	1.5 ± 1.4	2.3 ± 1.4 ***	3.0 ± 1.7	1.7 ± 1.3 ***
Vitamin E (mg/d)	30.5 ± 25.5	49.3 ± 16.6 ***	48.3 ± 19.1	37.2 ± 21.2 ***
Minerals				
Mg (mg/d)	395.4 ± 195.8	457.8 ± 123.5 ***	569.1 ± 161.9	393.0 ± 147.8 ***
Fe (mg/d)	16.5 ± 6.6	22.8 ± 7.6 ***	26.7 ± 8.3	17.8 ± 6.2 ***
Zn (mg/d)	5.7 ± 2.8	7.8 ± 2.3 ***	9.6 ± 3	6.0 ± 2.1 ***
Se (μg/d)	28.0 ± 14.9	32.4 ± 11.2 ***	42.7 ± 16.5	27.7 ± 11.6 ***
Caffeine (g/d)	1.2 ± 7.9	1.7 ± 8.7	1.4 ± 5.8	1.2 ± 6.3
Alcohol (g/d)	0.0 ± 0.3	0.0 ± 0.0 ***	0.0 ± 0.0	0.0 ± 0.2
Isoflavones (mg/d)	0.7 ± 0.5	1.1 ± 0.8 ***	1.1 ± 0.8	0.8 ± 0.6 ***
Anthocyanidins (mg/d)	37.6 ± 75.0	75.9 ± 90.8 ***	78.4 ± 95.9	50.3 ± 74.1 ***
Green/black tea (g/d)	3.3 ± 37.9	6.4 ± 43.2	4.2 ± 27.9	3.5 ± 30.3
Onion (g/d)	2.9 ± 8.8	10.1 ± 16.4 ***	12.5 ± 18.7	4.8 ± 9.5 ***
Garlic (g/d)	2.2 ± 4	5.7 ± 13.7 ***	5.4 ± 12.0	3.6 ± 8.3 **

rEDII, reverse energy-adjusted dietary inflammatory index; DDS, dietary diversity score; Q, quartile. Values are means ± standard deviations. *p* values are from generalized estimating equations adjusting for clustering by county. *** *p* < 0.001, ** *p* < 0.01.

**Table 4:** Associations of anti-inflammatory diet and dietary diversity during pregnancy with the incidence of depressive symptoms in rural China, 2015–2019.

	rEDII	Quartile of rEDII				DDS Groups	
		Q1	Q2	Q3	Q4	Adequate	Inadequate
EPDS scores Total n	2244	561	561	561	561	443	1801
Model 1 β (95% CI)	−0.25(−0.38, −0.13)	1.00(ref)	−0.43(−0.68, −0.18)	−0.59(−0.79, −0.39)	−0.53(−0.88, −0.18)	−0.38(−0.78, 0.03)	1.00(ref)
Model 2 β (95% CI)	−0.25(−0.38, −0.12)	1.00(ref)	−0.43(−0.68, −0.18)	−0.58(−0.79, −0.38)	−0.52(−0.88, −0.17)	−0.38(−0.78, 0.02)	1.00(ref)
Model 3 β (95% CI)	−0.25(−0.37, −0.12)	1.00(ref)	−0.41(−0.66, −0.15)	−0.58(−0.79, −0.37)	−0.50(−0.88, −0.13)	−0.39(−0.81, 0.03)	1.00(ref)
Depressive symptoms *n*/Total *n*	237/2244	71/561	56/561	59/561	51/561	38/443	199/1801
Model 1 RR (95% CI)	0.86(0.80, 0.92)	1.00(ref)	0.79(0.61, 1.01)	0.83(0.69, 1.00)	0.72(0.56, 0.92)	0.78(0.65, 0.93)	1.00(ref)
Model 2 RR (95% CI)	0.86(0.80,0.92)	1.00(ref)	0.80(0.62, 1.02)	0.84(0.69, 1.02)	0.72(0.57, 0.92)	0.77(0.65, 0.92)	1.00(ref)
Model 3 RR (95% CI)	0.87(0.81,0.93)	1.00(ref)	0.81(0.64, 1.03)	0.85(0.71, 1.02)	0.75(0.58, 0.97)	0.78(0.64, 0.95)	1.00(ref)

rEDII, reversed energy-adjusted dietary inflammatory index. DDS, dietary diversity score; EPDS, Edinburgh Postnatal Depression Scale; Q, quartile. Values are β or risk ratios (95% CI) for generalized estimating equations adjusted for clustering by country. Model 1: crude model. Model 2: adjusted for multivitamin supplement (folic acid, folic acid plus iron, folic acid plus B-complex vitamins); Model 3: Model 2 adjusted for maternal age (continuous), nulliparity status (nulliparity, multiparity), socioeconomic situation (lower, medium, upper), passive smoking (yes, no), and body mass index at enrollment (continuous).

**Table 5:** Interaction effect of anti-inflammatory diet with dietary diversity score on depressive symptoms in rural China, 2015–2019.

	β or RR (95% CI)	*p*
EPDS scores		
Interaction Model 1		
rEDII	−0.25(−0.33, −0.16)	<0.001
DDS group	−0.28(−0.61, 0.05)	<0.095
rEDII × DDS group	0.16(0.13, 0.19)	<0.001
Depressive symptoms		
Interaction Model 1		
rEDII	0.84(0.80, 0.89)	<0.001
DDS group	0.72(0.69, 0.75)	<0.001
rEDII × DDS group	1.39(1.11, 1.75)	0.004

EPDS, Edinburgh Postnatal Depression Scale. EDII, energy-adjusted dietary inflammatory index. rEDII, reversed EDII. DDS, dietary diversity score. β or RR were estimated using generalized estimating equations adjusted for clustering, multivitamin supplement (folic acid, folic acid plus iron, folic acid plus B-complex vitamins), maternal age (continuous), nulliparity status (nulliparity, multiparity), socioeconomic situation (lower, medium, upper), passive smoking (yes/no), and body mass index at enrollment (continuous).

**Table 6:** Stratified analysis of the association between anti-inflammatory diet and depressive symptoms according to dietary diversity adequacy status in rural China, 2015–2019.

	Total	Adequate DDS	Inadequate DDS
	β or RR (95% CI)	β or RR (95% CI)	β or RR (95% CI)
EPDS scores			
Total n	2244	443	1801
rEDII	−0.25(−0.37, −0.12)		
By DDS group		−0.10(−0.14, −0.05)	−0.24(−0.32, −0.16)
Depressive symptoms			
*n*/Total *n*	237/2244	38/443	199/1801
rEDII	0.87(0.81, 0.93)		
By DDS group		1.20(0.97, 1.50)	0.85(0.80, 0.90)

Edinburgh Postnatal Depression Scale, EPDS. EDII, energy-adjusted dietary inflammatory index. DDS, dietary diversity score. β or RR were estimated using generalized estimating equations adjusted for clustering, multivitamin supplement (folic acid, folic acid plus iron, or folic acid plus B-complex vitamins), maternal age (continuous), nulliparity status (nulliparity or multiparity), socioeconomic situation (lower, medium, or upper), passive smoking (yes/no), and body mass index at enrollment (continuous).

## Data Availability

The original contributions presented in the study are included in the article. Further inquiries can be directed to the corresponding author.

## Supplementary Materials

The following supporting information can be downloaded at https://www.mdpi.com/article/10.3390/nu17172823/s1, Figure S1: Study participant flow chart in rural China; Table S1: Food classification based on the Chinese Food Guide Pagoda (2022); Table S2: Food groups consumed by pregnant women in rural China by dietary diversity score.
