# Supplementary material for: Association Between Anti-Inflammatory Diet, Dietary Diversity, and Depressive Symptoms Among Chinese Pregnant Women
Source: Nutrients. 2025 Aug 29;17(17):2823. doi: 10.3390/nu17172823 (PMC12430108; doi:10.3390/nu17172823)
Supplement: Supplementary file 1 [file nutrients-17-02823-s001.zip › nutrients-3824715-supplementary.pdf]

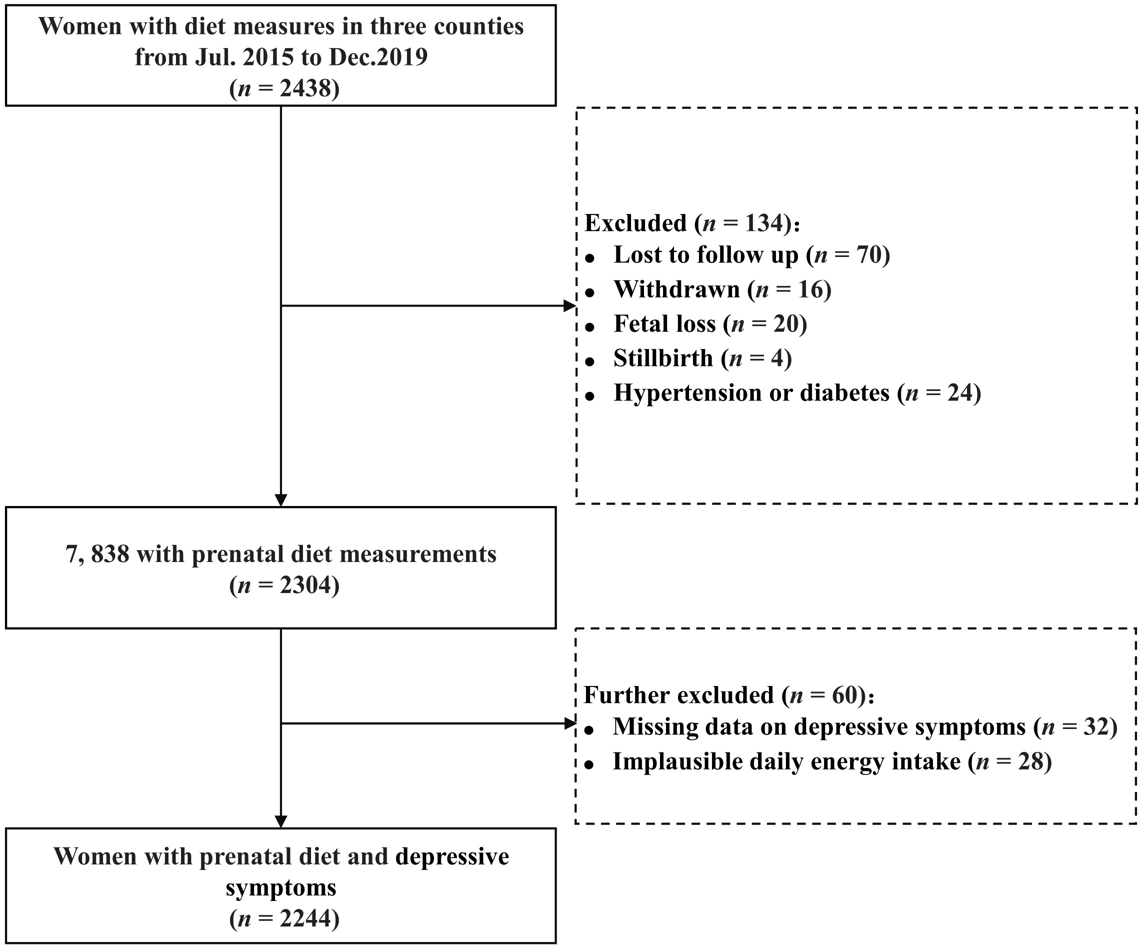

Figure S1. Study participant flow chart in rural China.

Table S1. Food classification based on the Chinese Food Guide Pagoda (2022).

Food groups	Food subgroups	Food items
Grains and tuber crop	Grains	Wheat noodle, instant soup noodle, wheat bun, wheat pancake, rice steamed, cold/rice noodles, dumpling, vegetable, dumpling, meat-filled, corn starch noodle, foxtail millet porridge, corn grits, bread, cookies
	Miscellaneous bean	Kidney bean, white, dried
	Tuber crop	Potato, white, sweet potato, red
Vegetables/fruit	Leafy green vegetables	Chilli or hot pepper, cucumber, bitter melon, zucchini, Chinese chive, cabbage, broccoli, spinach, water spinach, celery stem, romaine lettuce, Chinese cabbage, lettuce stem, bamboo shoot, scallion
	Dark yellow vegetables	Carrot, tomatoes, pumpkin
	Dark purple vegetables	Wood ear fungus, kelp, laver, eggplant
	Light white vegetables	Radish, winter melon, cauliflower, lotus root, onion bulb, needle mushroom, shitake mushroom, button mushroom
	Fruit	Apple, orange, banana, apricot/plum, pineapple, Hami melon, pomelo, watermelon, casaba, date, pear, grape, persimmon
Animal-based food	Red meat	Pork, lean and fat, beef, lean and fat, lamb, lean and fat
	White meat	Poultry
	Organ meat	Pork, liver, pork, blood, pork, kidney, beef, kidney
	Processed meat	Pork, becon, pork sausage/cured sausage, pork, ham sausage
	Fish/seafood	Fresh-water fish, saltwater fish, dried shrimp, fresh shrimp or crab
	Whole eggs	Eggs, duck/quail eggs
Milk-based/(legumes and nuts)	Milk and dairy products	Milk, powdered milk, yoghurt
	Legumes and nuts	Soybean, tofu, soybean curd, soybean milk, soybean curd slab, semisoft, beans/cowpeas/ green beans, sprout, mung bean, walnut, peanut, watermelon seed
Condiments	Vegetable oils	Vegetable oils
	Animal oils	Animal oils
	Salt	salt
	White/brown sugar	White/brown sugar
	Soy sauce	Soy sauce
Snacks/drinks	Snacks	Ice cream, rice crust/twist, candy, crystal cake, cake or cupcake, chocolate
	Soft drinks	Tea, coffee, soda drink, juice
	Alcohol drinks	Beer, Baijiu, wine

Table S2. Food groups consumed by pregnant women in rural China by dietary diversity score.

Food groups	Food items	Overall	Dietary Diversity Score	
			Adequate	Inadequate
			n=443	*n*=1801
Starchy staples	Wheat noodle, instant soup noodle, wheat bun, wheat pancake, rice steamed, cold/rice noodles, vegetarian dumplings, meat dumplings, rice noodles, millet gruel, corn grits, rice crust/twist, bread, Chinese snacks, biscuits, potatoes, sweet potatoes	2204(98.2)	442(19.7)	1763(78.6)**
Beans and peas	Soybeans, other beans, tofu, tofu pudding, soybean milk, dried tofu, green bean, mung bean sprout	253 (11.3)	182(8.1)	71(3.2)***
Nuts and seeds	Walnuts, roasted peanuts, sunflower seeds	38 (1.7)	24(1.1)	14(0.6)***
Dairy	Whole cow's milk, cow's milk powder, yogurt	468 (20.9)	205(9.1)	263(11.7)***
Flesh foods (meats)	Fresh fatty/lean pork, beef, mutton, chicken/duck meat, cured meat, sausage, ham sausage, liver, blood, kidney, other animal offal, freshwater fish, dried shrimp skin, fresh shrimp	34 (1.5)	30(1.3)	4(0.2)***
Eggs	Eggs	1367(60.9)	379(16.9)	988(44.0)***
Vitamin A-rich dark green vegetable	Fresh pointed pepper, cucumber, bitter melon, zucchini, Chinese chives, cabbage, broccoli, spinach, water spinach, celery, romaine lettuce, lettuce, bamboo shoots, green onion	717(32.0)	370(16.5)	347(15.5)***
Other vitamin A-rich fruits and vegetables	Carrot, tomato, Hami melon, pumpkin, dried nori, tangerine, plum/apricot, watermelon, muskmelon, fresh jujube, persimmon, Chinese cabbage	1277(56.9)	428(19.1)	849(37.8)***
Other vegetables	Soaked black fungus, soaked kelp, eggplant, white radish, lotus root, onion, garlic, enoki mushrooms, shiitake mushrooms, mushrooms	111(5.0)	98(4.4)	13(0.6) ***
Other fruits	Apple, banana, pineapple, grapefruit, pear, grape, fruit juice beverage	764(34.1)	347(15.5)	417(18.6)***

Values are *n* (%).Tests of significance are adjusted for clustering by county using a generalized estimating equation. ***$p < 0.001$, **$p < 0.01$.
